# Companionship With Family, Friends, and Neighbors in Later Life

**DOI:** 10.1093/geroni/igaa057.1306

**Published:** 2020-12-16

**Authors:** Ikuko Sugawara, Midori Takayama, Yoshiko Ishioka

**Affiliations:** 1 University of Tokyo, Tokyo, Japan; 2 Keio University, Yokohama, Japan

## Abstract

Companionship with close others are known to have a significant positive effect on our well-being in later years of life. At the same time, it is known that the frequency of meeting and chatting with others, an indicator of companionship, declines as we age. In this study we explore the situation of companionship among older-old and oldest-old people. The focus of this study on understanding how the aging process affects the experience of companionship and how people adapt to the loss of companionship. Semi-structured, in-depth interviews with 43 people aged 75 and older living in urban communities in Kawasaki, Japan. Participants were asked about everyday interactions with close others and feelings they experienced at the time. Interviews were transcribed and analyzed qualitatively. Chatting, going out for lunch or dinner, and going shopping were examples of activities older Japanese enjoyed with close others. Almost all respondents mentioned the loss of their old friends and siblings. They also mentioned that the decline in their physical and cognitive health, as well as that of their companions hindered shared activities they used to enjoy. They cherished positive interactions with others, although the frequency declined. Some respondents intentionally made new companions in the physical proximity, but it was hard to compensate for the loss of old companions with new one. These findings suggest that the value of companionship remains or even increase as we age. It would be important to identify environmental or social factors that may prevent the loss of companionship among older adults.

